# Anti-Inflammatory and Joint-Protective Effects of Blueberries in a Monosodium Iodoacetate (MIA)-Induced Rat Model of Osteoarthritis

**DOI:** 10.3390/nu17132134

**Published:** 2025-06-27

**Authors:** Sanique M. South, Keith Crabtree, Dayna L. Averitt, Parakat Vijayagopal, Shanil Juma

**Affiliations:** 1 Phil and Penny Knight Campus for Accelerating Scientific Impact, University of Oregon, Eugene, OR 97403, USA; ssouth@uoregon.edu; 2 Department of Nutrition and Food Science, Texas Woman’s University, Denton, TX 76204, USA; kcrabtree@twu.edu (K.C.); parakatv@gmail.com (P.V.); 3 Division of Biology, Texas Woman’s University, Denton, TX 76204, USA; daveritt@twu.edu

**Keywords:** blueberries, inflammation, pain, knee osteoarthritis, phytochemicals, bioactives

## Abstract

Background/Objectives: Osteoarthritis is a degenerative joint disease that affects people worldwide. It is characterized by joint pain, synovial inflammation, and the degradation of articular cartilage with subchondral bone. Presently, there is no known cure, and pharmacological treatment options are limited. Blueberries contain phytochemicals, which have been linked to positive health benefits and may offer therapeutic benefits. Therefore, the purpose of this study was to examine the dose-dependent effects of whole blueberries on arthritis symptoms in a monosodium iodoacetate (MIA)-induced rat model of osteoarthritis. Methods: Forty female rats were used for this study. Thirty were injected with MIA to induce joint degradation associated with osteoarthritis. Ten served as controls without MIA induction. The MIA-induced rats were randomized into three groups. All groups were fed a casein-based diet, with two of the MIA-induced groups receiving an addition of whole-blueberry powder at 5% and 10%. Fasted blood specimens and tissues of interest were collected post-euthanasia for analysis. Mechanical allodynia and joint widths were assessed throughout this study. Results: MIA induction resulted in changes in pain behaviors and the development of mechanical allodynia. The MIA injection produced inflammation, pain symptoms, and behaviors that are representative of those observed in humans with osteoarthritis. The incorporation of whole blueberries into diets reduced pain behaviors and inflammation. Conclusions: Overall, whole blueberries showed limited, non-dose-dependent effects in the MIA-induced rat model of osteoarthritis. While some outcomes improved in blueberry-treated groups, the overall results were not consistently significant. These preliminary findings suggest potential biological activity and support the further investigation of blueberries’ therapeutic efficacy.

## 1. Introduction

Osteoarthritis is the most common chronic joint disease that affects approximately 300 million people worldwide [1]. In the United States, osteoarthritis affects approximately 30.8 million adults and is the leading cause of disability [1,2,3]. Around 22.7 million or more people in the United States report arthritis-related limitations in performing activities [4]. Ma and colleagues have reported that approximately 80% of adults affected by osteoarthritis will experience some amount of mobility limitations [5]. The primary symptoms of osteoarthritis include chronic pain, synovial inflammation, bone remodeling, erosion of the articular cartilage, and stiffness of the joints. Previously, osteoarthritis was mainly linked to aging. However, research has advanced, and its etiology is presently considered multifactorial and linked to various risk factors [6]. The major risk factors of osteoarthritis include, but are not limited to, age and obesity. As a result of the rising trends in obesity and the aging population, the incidence and prevalence of osteoarthritis are expected to grow rapidly in the next decade [4]. The risk factors of osteoarthritis are classified as non-modifiable, such as gender and genetics, or modifiable, such as obesity [1,6]. Females are disproportionately more susceptible to osteoarthritis development, and they present more severe osteoarthritis when compared to males [3]. Given the sex-specific differences, the present study utilized female rats to better model the biological characteristics of osteoarthritis in this at-risk population. Notwithstanding the younger age of the rats in this study, female samples allow for an initial investigation of female-specific responses to dietary interventions.

The pathogenesis and progression of osteoarthritis are linked to inflammation [1,7]. In healthy cartilage, there is a balance between synthesis and degradation. However, in osteoarthritis, degradation is favored over synthesis [8,9]. During the progression of osteoarthritis, the body’s immune system increases the production of inflammatory cytokines [1]. These cytokines contribute to cartilage destruction and disease progression [1]. Two main inflammatory cytokines linked to the progression of osteoarthritis are interleukin-1 (IL-1) and tumor necrosis factor alpha (TNFα) [9]. The increased production of IL-1 beta (IL-1β) and TNFα stimulates the production of other proinflammatory cytokines, such as IL-6, IL-8, IL-11, and IL-17, thus contributing to continued degradative activity [9,10]. In addition, anti-inflammatory cytokines such as IL-10 and IL-13 are also released in response to the degradative activities. Interleukin (IL)-10 increases the synthesis of type II collagen and aggrecan; inhibits the synthesis of metalloproteinases (MMPs) and apoptosis of chondrocytes; and stimulates the production of IL-1β antagonists, such as the tissue inhibitors of matrix metalloproteinases (TIMP-1) and growth factors [11]. Disease progression favors the degradative process, and anti-inflammatory mediators prove less effective [9]. Additionally, in osteoarthritis, there are increased levels of proteolytic enzymes, such as MMPs and aggrecanases. The MMPs degrade proteoglycans and collagen, while aggrecanases degrade aggrecan [8,10]. TIMPs regulate MMPs, and cartilage degradation by MMPs during osteoarthritis is linked to decreased levels of TIMPs [10,12]. Research states that inhibiting the expression of specific MMPs limits the degradation of articular cartilage [8]. However, the etiology of osteoarthritis remains unresolved, and as a result, there is no known cure for osteoarthritis [1,2,13].

Treatment options for osteoarthritis are categorized into non-pharmacological, pharmacological, or surgical options [1]. These options are aimed at relieving the symptoms of osteoarthritis and slowing the progression of the disease [1,10]. The main pharmacological treatment options for osteoarthritis include non-steroidal anti-inflammatory drugs (NSAIDs) and acetaminophen [1]. Compared to NSAIDs, acetaminophen is less effective [1]. However, prolonged NSAID use may result in severe gastrointestinal disturbances and renal failure [13,14]. Therefore, there is increased interest in the use of non-pharmacological treatment options to treat osteoarthritis [15]. Non-pharmacological approaches for the treatment of osteoarthritis include diet and exercise [2]. Non-pharmacological treatments present positive health benefits with little to no adverse side effects compared to pharmacological treatment options.

Dietary fruits such as blueberries, raspberries, and strawberries contain a rich source of phytochemicals and nutrients, which contribute to their anti-inflammatory and antioxidant properties [2]. Blueberries contain phytochemicals such as polyphenols, which are linked to their antioxidant properties [16]. Figueira et al. (2016), in an in vivo model of arthritis, reported reduced paw edema, osteophyte formation, and bone resorption in animals treated with blueberry extracts [16]. Similarly, research studies highlight the promising health benefits of whole-blueberry supplementation in metabolic syndrome, memory, neuronal aging, and osteoporosis [17,18,19,20,21]. Therefore, whole-blueberry supplementation may exhibit anti-inflammatory and joint-protective effects in diseases such as osteoarthritis. The purpose of this study was to investigate the anti-inflammatory and joint-protective effects of whole blueberries using a monosodium iodoacetate (MIA)-induced rat model of osteoarthritis.

## 2. Materials and Methods

### 2.1. Animals

A total of forty 45-day-old female (initial weight of 140–160 g) CD rats were obtained from Charles River Laboratories (Wilmington, MA, USA) and used for this study. Thirty rats were injected with monosodium iodoacetate (MIA) into the right knee joint to induce osteoarthritis. Ten rats served as a control group without the induction of osteoarthritis (did not receive an injection). An experienced veterinarian at Charles River Laboratories (Wilmington, MA, USA) performed the injection prior to shipping. Briefly, the rats were anesthetized using isoflurane gas, and the right knee was prepped aseptically. The hair was shaved, and the skin was sanitized under sterile conditions. Following this step, 3 mg of MIA (Mono-Iodoacetate) in 50 µL of sterile 0.9% saline was injected through the patellar tendon into the knee joint cavity. The rats were recovered from anesthesia and managed postoperatively until they were shipped. Upon arrival at the TWU animal facility, the rats were individually housed in cages on a 12 h light and 12 h dark cycle with access to food and water ad libitum. The rats were maintained in a temperature-controlled vivarium and allowed to acclimatize for one week before testing. The MIA-induced rats were weight-matched and randomly assigned to three study groups. All MIA groups and control rats received a casein-based diet (Envigo Teklad, Madison, WI, USA), with two groups receiving an addition of freeze-dried blueberries (Envigo Teklad, Madison, WI, USA). The experimental groups of the present study included the following: (1) non-MIA (control, no blueberries added; N = 10); (2) MIA—0% BB (no blueberries added; N = 10); (3) MIA—5% BB (5% blueberries added to diet; N = 10); and (4) MIA—10% BB (10% blueberries added to diet; N = 10). The animals were weighed weekly throughout the study period, and food intake was monitored and recorded. At the end of the study, following an overnight fast, the animals were anesthetized with 3–5% isoflurane. Blood was collected (4–8 mL) from the posterior aorta in vacutainer tubes (BD Biosciences, San Jose, CA, USA). Blood serum was separated using centrifugation (~1500–2000× *g* for 15 min) and stored at −80 °C within an hour for later analysis. Following blood collection, the animals were euthanized via the transection of the posterior aorta. The right and left knee joints were obtained, and the cartilage tissue was collected for protein lysate and gene analysis. All experimental procedures were performed under the approval of the Texas Woman’s University Institutional Animal Care and Use Committee (approved by IACUC; protocol number 2019-01). The experiments conformed to federal guidelines and the Committee for Research and Ethical Issues of the International Association for the Study of Pain.

### 2.2. Treatment

The animals’ initial body weights were recorded after one week of acclimatization. The 30 MIA-induced animals were randomly assigned to three groups consisting of 10 animals based on equal body weights. This study’s treatment began with the animals in group 1 (non-MIA) and group 2 (MIA) being fed a casein-based diet (AIN-93M, Envigo Teklad, Madison, WI, USA), and group 3 and group 4 were fed a casein-based diet (AIN-93M, Modification, Envigo Teklad, Madison, WI, USA) with the addition of whole-blueberry powder at 5 and 10 percent, respectively (Table 1). The justification for the dose of blueberries is based on previous animal studies conducted in an ovariectomized rat model of bone loss [21,22]. The treatment schedule was initiated using a staggered schedule that allowed ten animals to be euthanized per day over a four-day period. All groups were fed the respective diets for 48 days.

### 2.3. Behavioral and Edema Analysis

A total of six animals from each treatment and control group, for a total of 24 animals, were randomly selected based on the staggered treatment and tested using the Dynamic Plantar Aesthesiometer Test, as previously described [23]. Due to limitations in available resources, it was not feasible to test all 10 samples per group. Therefore, a representative subset was selected prior to the start of testing to ensure consistency and minimize sampling bias across all experimental groups. This sample size was determined to be sufficient for detecting meaningful changes while maintaining scientific rigor. The same 24 animals were tested at each time period. The presence of mechanical allodynia in the hind paw of the affected joint was confirmed prior to the initiation of treatment and then reassessed on days 13, 26, and 40 of the treatment. The animals were acclimatized to the behavior testing room 24 h prior to testing in a quiet environment. Each animal was placed in a clear, non-restraining Plexiglass box on an elevated grid platform, and a blunt, non-noxious mechanical probe was aimed at the plantar surface of the hind paw of the arthritic joint. The force of the mechanical probe was increased with a ramp of 3 g per second over 10 s, with a cutoff of 30 s to avoid mechanical lifting of the paw by the device. Increased sensitivity was noted as a decrease in the grams of force required to elicit a paw withdrawal. Three readings were recorded and averaged, and the data were analyzed. Edema testing was also conducted on the 24 animals on the same days that they were assessed for mechanical allodynia. The thickest point of the arthritic knee joints was measured using a digital caliper. The measurements were recorded, and the data were analyzed.

### 2.4. Tissue Collection and Analysis

After the 48-day treatment period, following an overnight fast, the animals were anesthetized with 3–5% isoflurane. Blood was collected (4–8 mL) from the posterior aorta in vacutainer tubes (BD Biosciences, San Jose, CA, USA). Blood serum was separated using centrifugation (3000 rpm for 15 min) and stored at −80 °C within an hour for later analysis. Following blood collection, the animals were euthanized via the transection of the posterior aorta. The right and left knee joints were obtained, and the cartilage tissue was collected for gene analysis.

### 2.5. Serum Inflammatory and Cartilage Markers

Serum was separated using centrifugation and stored at −80 °C within an hour for later analysis. The levels of TIMP-1 and IL-1β in the serum were assessed using a multiplex panel (Rat Cytokine Array Kit; R&D Systems; Minneapolis, MN, USA) according to the manufacturer’s protocol. The levels of IL-10 in the serum were analyzed using a Quantikine ELISA Kit from R&D Systems, according to the manufacturer’s protocol (Minneapolis, MN, USA). The analysis of hyaluronic acid in rat serum was conducted using ELISA kits acquired from Quidel, Inc. (San Diego, CA, USA).

### 2.6. Western Blot Analysis

The right knee joint was excised and cleaned of soft tissue, and the patella was exposed to obtain cartilage tissue. The cartilage was collected, snap-frozen in liquid nitrogen, and then stored at −80 °C for later analysis. The cartilage tissue was homogenized to obtain cell lysates using RIPA lysis and an extraction buffer (89901; Thermo Fisher Scientific, Waltham, MA, USA). Protein concentrations were determined using the Pierce bicinchoninic acid (BCA) protein assay kit (Rockford, IL, USA). The total protein (40 µg) in the rat samples was resolved in 10% sodium dodecyl sulfate-polyacrylamide gel electrophoresis (SDS-PAGE) and electroblotted onto a nitrocellulose membrane. The equal transfer of proteins was confirmed by Ponceau S staining (Bio-Rad Laboratories, Hercules, CA, USA). Following staining, the membrane was blocked with a blocking buffer (5% nonfat dry milk in TBS buffer) for one hour. After, the membrane was washed three times in TBST and then incubated with NFκB and cartilage oligomeric protein (COMP) antibodies, along with β-actin antibodies, as housekeeping protein markers overnight at 4 °C with the dilutions proposed by the manufacturers (Cell Signaling Technology, Inc., Danvers, MA, USA; Abcam, Cambridge, MA, USA). After overnight incubation, the membrane was washed with TBS and Tween 20 and then incubated with IRDye secondary antibodies (Li-Cor, Lincoln, NE, USA) in blocking buffers for one hour at room temperature. The protein bands were visualized using a chemifluorescent imager (Li-Cor, Lincoln, NE, USA). Band intensities were quantified using Image StudioTM software version 3.1 and normalized versus β-actin as an internal control for total protein loading. The relative protein expression levels were expressed as a ratio of band intensities to β-actin.

### 2.7. Gene Expression Analysis

Gene expression was examined by extracting total RNA from homogenized cartilage tissues using the Qiagen Plus Universal mini Kit according to the manufacturer’s protocol (Germantown, MD, USA), and the expression was analyzed and quantified. The concentration and purity of the total RNA were determined by measuring the optical density at 260 nm and 280 nm. The isolated total RNA was diluted and stored in aliquots at −80 °C. Reverse transcription was performed using the iScript reverse transcription Supermix kit (BioRad, Hercules, CA, USA) and according to the manufacturer’s instructions. The cDNA samples were used in triplicate for the real-time PCR. The relative expression of Spp1, Pten, Ptgs2, Tlr3, MMP9, Sox9, Birc5, Tnf, Smad7, Tgfb2, MMP2, Sirt1, Kdr, Sulf1, Smad3, Col1a2, Tnfrsf1, Col1a1, IL6, and GAPDH was measured by real-time PCR using the SsoAdvanced Universal SYBR Green Supermix (BioRad) with a PrimePCR customized plate (BioRad) containing a specific primer. All PCR samples went through several cycles at varying annealing temperatures to enable denaturation, extension, and elongation. The relative gene expression was calculated by normalizing their expression levels to that of the glyceraldyhyde-3-phosphate dehydrogenase (GAPDH) gene (internal control).

### 2.8. Histopathological Analysis

The right knee joints of five randomized animals from each group were excised and fixed in 10% neutral buffered formalin for 72 h at 4 °C. The samples were decalcified in 5% formic acid for 3 days, after which they were embedded in paraffin, sectioned using a microtome, and stained. Sections of 6 µm thickness were made and used for hematoxylin and eosin staining. The stained joint sections were examined and scored for inflammatory infiltration, synovial hyperplasia, and the erosion of the cartilage and bone [24,25]. Each joint section was scored on a scale of 0 to 4 (Table 2) according to previous studies [24,25]. A scientist who was blinded to the treatment group for the joint sections performed the histopathological joint scoring.

### 2.9. Statistical Analysis

Descriptive statistics were calculated for all variables and include mean, standard deviation, and upper and lower limits. Blood analysis data are expressed as a group mean + standard error of mean (SEM) of the concentration of the markers assessed. Gene expression was measured in cartilage samples and expressed relative to GAPDH. The difference between treatment groups was assessed using *t*-test analysis, one-way analysis of variance (ANOVA), and repeated measures ANOVA, along with Tukey’s post hoc test. Histology scores were analyzed using a Kruskal–Wallis test, with Dunn’s test used for post hoc analysis. Samples were run in triplicate for the RT-PCR analysis and in duplicate for all other experiments. A *p* value of ≤0.05 was considered significant. All statistical analyses were performed using IBM SPSS statistics version 25 or GraphPad Prism version 9.

## 3. Results

### 3.1. Effect of Blueberry Diet on Body Weight

**Figure 1:**
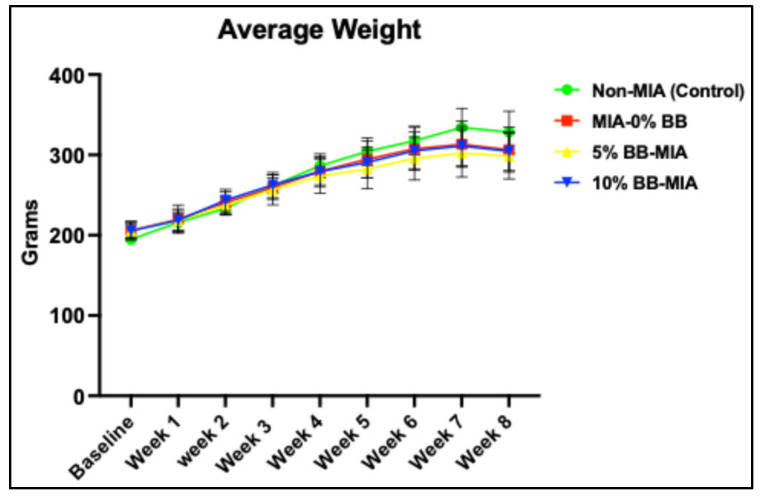
Effects of whole-blueberry treatment on body weight in control and MIA-induced rats. Average weight throughout the study period from baseline to final period. Data presented as ±SD. There was no difference in body weights between the four groups at baseline and the final period. N = 10 for each treatment group.

All rats survived and tolerated the intervention. At baseline, there were no significant differences in weight between the four groups (Figure 1). There was a consistent increase in the average weight for animals in all groups throughout this study. There was no difference in body weights between the four groups at the end of the treatment period (*p* ≤ 0.05). The induction of MIA-induced osteoarthritis in rats had no effect on the average weight.

### 3.2. Pain and Edema

**Figure 2:**
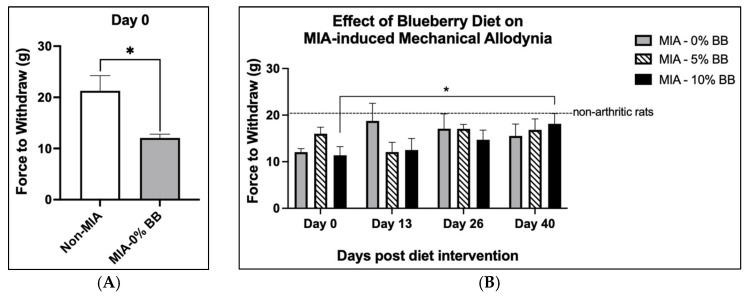
Effects of whole-blueberry treatment on mechanical allodynia in MIA-induced rats: (**A**) Mechanical allodynia was significantly higher at baseline (day 0) in the MIA group in comparison to the control. N = 6 for the control group and N = 18 for the MIA group. (**B**) Mechanical allodynia was significantly reduced in the MIA + 10% BB group at day 40 compared to baseline day 0, whereas, in the MIA—5% BBP group and the MIA—0% BBP group, there was no difference in mechanical allodynia at day 40 in comparison to day 0. N = 6 in each group. Asterisk (*) denotes statistical significance (*p* ≤ 0.05) in comparison to the control group. The results shown are expressed as mean ± SEM, *p* ≤ 0.05.

Animals were injected with monosodium iodoacetate (MIA) to induce osteoarthritis. At baseline, before diet intervention, mechanical allodynia was significantly higher in the MIA group compared to the control group (Figure 2A). The results indicated that the induction of osteoarthritis in animals with MIA injections was successful, as evidenced by pain behaviors such as mechanical allodynia in the MIA group and not in the control group. In addition, animals were examined at three other time points throughout this study to assess mechanical allodynia. Mechanical allodynia was significantly lowered at day 40 compared to day 0 in the MIA + 10% BB group (Figure 2B). This was evident as there was an increased threshold in the grams of pressure needed to elicit a paw withdrawal at day 40 compared to day 0. At day 40, there was a trend showing higher mechanical allodynia in the MIA group compared to the MIA + 5% BB and MIA + 10% BB groups (but not significant). There was no significant difference from day 0 to day 40 in the MIA + 0% BB and MIA + 5% BB groups. The addition of blueberries into the diets of the animals significantly (*p* ≤ 0.05) reduced pain behaviors in the MIA + 10% BB treatment group in comparison to the MIA group.

Edema in the joints was assessed by examining the joint width of the animals throughout this study. The MIA injections were made approximately 10 days prior to baseline assessment. There was no significant (*p* ≤ 0.05) difference at baseline between the MIA group and the two treatment groups, as seen in Table 3. Within each group, there was no significant (*p* ≤ 0.05) difference from day 0 to day 40.

### 3.3. Effects of Blueberry Diet on Serum Inflammatory and Cartilage Markers

**Figure 3:**
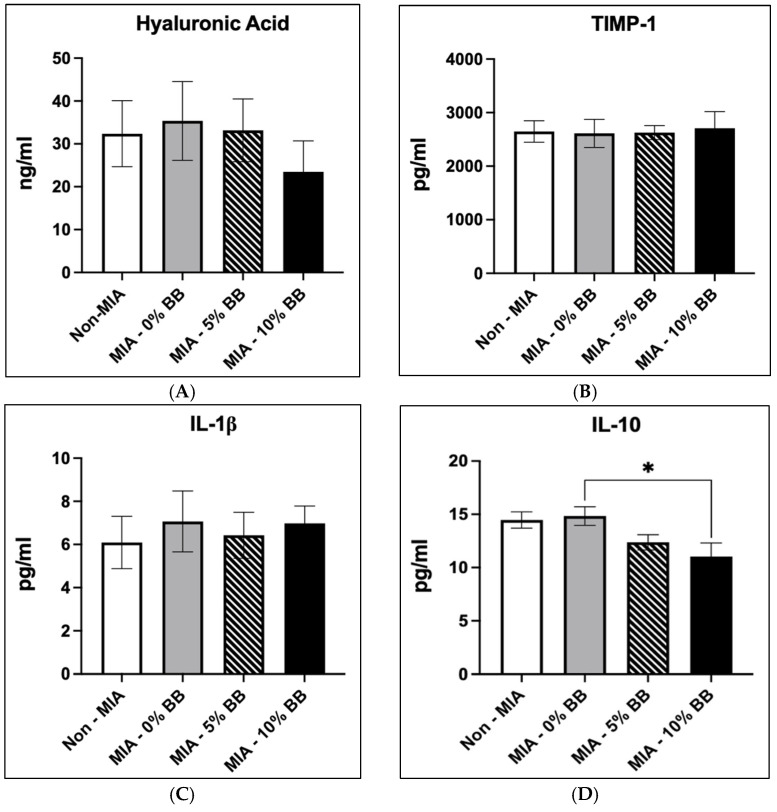
Effects of whole-blueberry treatment on the following: (**A**) hyaluronic acid; (**B**) tissue inhibitor of metalloproteinases (TIMP-1); (**C**) IL-1β and (**D**) IL-10 concentrations in blood samples. N = 10 for each treatment group. The results shown are expressed as mean ± SEM, *p* ≤ 0.05. Asterisk (*) indicates significance of *p* ≤ 0.05.

We examined the effects of the blueberry-supplemented diet on serum inflammatory and cartilage markers. The effect of the blueberry diet on hyaluronic acid concentrations is shown in Figure 3A. There was no difference in hyaluronic concentration in the MIA group compared to the control group. Blueberry diet had no effect on the concentration of hyaluronic acid in both the 5% BB-MIA and the 10% BB-MIA groups compared to the MIA group.

The results showed that TIMP-1 was not statistically different in the MIA group compared to the control group, as shown in Figure 3B. There was no difference in the concentration of TIMP-1 in the blueberry treatment groups in comparison to the MIA group. The effects of the blueberry diet on IL-1β concentrations are shown in Figure 3C. The MIA group reported a higher concentration of IL-1β in comparison to the control group, but it was not significant. There was no difference in IL-1β concentrations in the blueberry treatment groups and the MIA group. There was no significant difference in IL-10 concentrations in the MIA group in comparison to the control (Figure 3D). However, treatment with whole blueberries dose-dependently decreased the concentration of IL-10 compared to the MIA group (Figure 3D). The concentration of IL-10 was significantly lower (*p* ≤ 0.05) in the 10% BB-MIA treatment group compared to the MIA group.

### 3.4. Protein Expression

**Figure 4:**
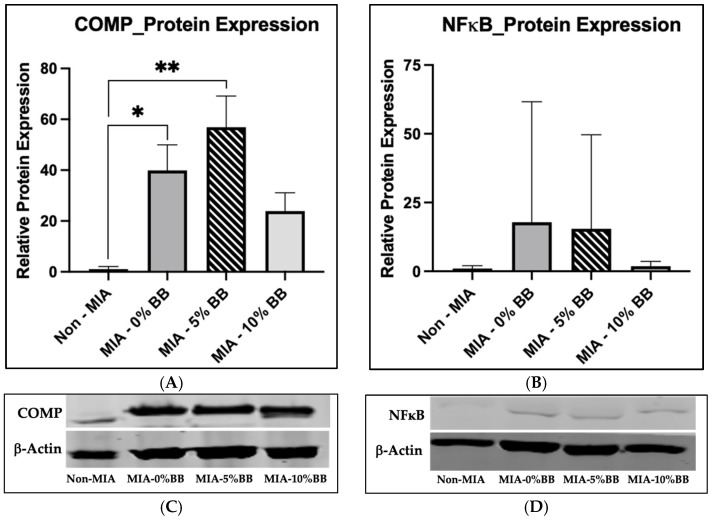
Effects of whole-blueberry treatment on protein expression in MIA-induced rats. Western blot analysis of (**A**,**C**) COMP and (**B**,**D**) NFκB protein expression in cartilage samples. The results shown are expressed as mean ± SEM, *p* ≤ 0.05. Asterisk (*) indicates significance of *p* ≤ 0.05 and double asterisk (**) indicates significance of *p* ≤ 0.01.

To determine the effect of whole blueberries on protein expression in an MIA-induced model, we performed Western blot analysis. The protein expression levels of cartilage oligomeric protein (COMP) and nuclear factor-kappa B (NFκB) were examined. The expression of COMP was significantly increased in the MIA group compared to the control group (Figure 4A,C). The COMP protein expression was significantly higher in the 5% whole-blueberry treatment compared to the control. The addition of whole blueberries did not affect the protein expression of COMP compared with the MIA group (Figure 4A). Interestingly, there was no difference between the control and 10% BB groups’ protein expressions. The expression of COMP in the 10% whole-blueberry-treated group was lower compared to the MIA group, but it was not statistically significant. In addition, the expression of NFκB was greater in the MIA group compared to the control (Figure 4B,D), but it was not statistically significant. The treatment of the MIA-induced rats with whole blueberries at 5% and 10% had no effect on NFκB protein expression compared to the MIA group. It should be noted that the 5% and 10% whole-blueberry treatment groups’ protein expressions were trending lower compared to the MIA group.

### 3.5. Effect of Blueberry Diet on Gene Expression

**Figure 5:**
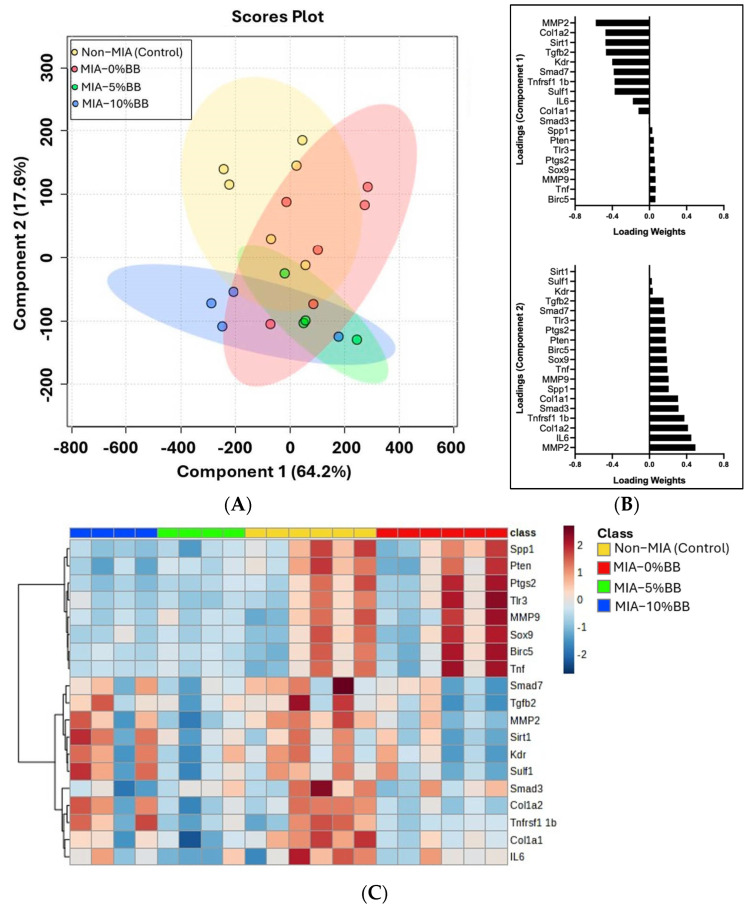
The effects of whole-blueberry treatment on gene expression in MIA-induced rats were analyzed using partial least squares analysis (PLSDA) and hierarchical clustering. The results of the PLSDA plot are represented by (**A**) the 2-D score plots of Component 1 and Component 2 and (**B**) the loading plot representing Component 1 and Component 2. Hierarchical clustering results are shown as a (**C**) heatmap. The darker red color indicates higher expression, and the blue color indicates lower expression.

To evaluate the effect of whole-blueberry supplementation on gene regulation, we quantified the expression of secreted phosphoprotein (Spp1), phosphatase and tensin homolog (Pten), prostaglandin-endoperoxide synthase 2 (Ptgs2), Toll-like receptor 3 (Tlr3), MMP9, SRY-Box transcription factor 9 (Sox9), baculoviral repeat-containing 5 (Birc5), Tnf, mothers against decapentaplegic homolog 7 (Smad7), transforming growth factor beta-2 (Tgfb2), MMP2, sirtuin 1 (Sirt1), kinase insert domain receptor (Kdr), sulfatase (Sulf1), Smad3, collagen type 1 alpha 2 chain (Col1a2), TNF receptor superfamily member 1 (Tnfrsf1), Col1a1, IL6, and glyceraldehyde-3-phosphate dehydrogenase (GAPDH) in cartilage samples. Surprisingly, the results from the partial least squares analysis (PLSDA) score plots (Figure 5A) showed that there was no complete separation between the treatment groups, with overlaps observed. Component 1 accounted for 64.2% of the variance and was largely responsible for group discrimination. While Component 2 accounted for 17.6% of the variance. The genes associated with each component driving the discrimination between the treatment groups are shown in the loading plots of Component 1 and Component 2 (Figure 5B). The hierarchical cluster heatmap analysis of 19 genes revealed various clusters, but there were two distinct clusters of genes that were observably different (Figure 5C). The expression of eight genes was higher in the MIA group compared to the control and whole-blueberry treatment groups. These genes were Spp1, Pten, Ptgs2, Tlr3, MMP9, Sox9, Birc5, and Tnf. Also, the expression of 11 genes was lower in the MIA group compared to the control group. These were Smad7, Tgfb2, MMP2, Sirt1, Kdr, Sulf1, Smad3, Col1a2, Tnfrsf1, Col1a1, and IL6. The heatmap demonstrated that genes with higher expression in the MIA group are genes related to inflammation (Tnf), chondrogenesis (Sox9), and antiapoptotic pathways (Pten). Comparatively, genes with lower expression in the MIA group were related to extracellular matrix (ECM) remodeling (MMP2), ECM components (Col1a1), VEGF receptor (kdr), and TGF-beta signaling (Smad3). Whole blueberries may be effective in modulating the gene expression of key inflammatory mediators and other signaling pathways. Nevertheless, further research is warranted.

### 3.6. Effects of Blueberry Diet on Joint Tissue

**Figure 6:**
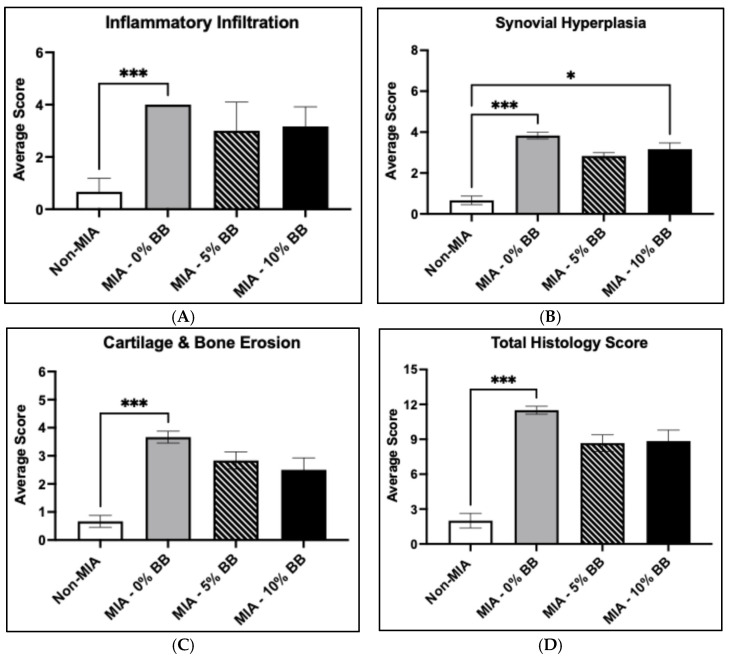
Effects of whole-blueberry treatment on MIA-induced rats. Stained joint sections were examined and scored for (**A**) inflammatory infiltration, (**B**) synovial hyperplasia, (**C**) erosion of the cartilage and bone, and (**D**) total histology score of the joints. N = 5 for each treatment group. Within the MIA—0% BBP group, all animals received a similar score for inflammatory infiltration, resulting in a SEM score of zero; hence, no error bar is displayed for that group. Asterisk (*) denotes statistical significance (*p* ≤ 0.05) in comparison to the MIA group. The results shown are expressed as ± SEM, *p* ≤ 0.05. Triple asterisk (***) indicates significance of *p* ≤ 0.001.

The effects of whole blueberries on cartilage damage were evaluated in the MIA-induced rats. Knee joint sections were stained, examined, and scored based on inflammatory infiltration, synovial hyperplasia, and cartilage and bone erosion. For all parameters, the control group showed intact cartilage structure. However, compared to the control group, the MIA—0%BB group showed increased inflammatory infiltration, increased synovial hyperplasia, and severe erosion of the cartilage and bone relative to the control non-MIA group (Figure 6). In the 5% and 10% whole-blueberry treatment groups, inflammatory infiltration was observably lower compared to the MIA group, but it was not statistically significant (Figure 6A). Comparably, synovial hyperplasia was less prevalent in the 5% whole-blueberry group and trended lower in the 10% whole-blueberry group compared to the MIA group (Figure 6B). However, synovial hyperplasia was significantly increased in the MIA—10%BB group compared to the non-MIA group. The cartilage structure in the whole-blueberry-treated groups was closer to that of the control. The degeneration observed in the blueberry groups was not as extensive as that of the MIA group, which had significantly more degradation compared to the non-MIA group (Figure 6C). Overall, the total histology score was significantly higher in the MIA group compared to the control group (Figure 6D). Also, the total histology scores indicated increased structural damage in the MIA group compared to the treatment groups. The addition of whole blueberries inhibited structural damage compared to the MIA group with no treatment.

## 4. Discussion

The present study evaluated the anti-inflammatory and joint-protective effects of whole blueberries using a monosodium iodoacetate (MIA)-induced rat model of osteoarthritis. MIA induction in animals has been shown to produce features similar to those observed in the clinical conditions of osteoarthritis [26]. The MIA induction inhibits glycolysis, which produces changes in the articular cartilage and, as a result, produces cartilage destruction representative of that seen in human osteoarthritis [26,27,28]. In addition, the MIA animal model mimics the pain symptoms comparable to those observed in human osteoarthritis conditions [29]. This model is well established and effective in investigating the effects of complementary treatment modalities for osteoarthritis. The addition of whole blueberries into the diet may provide therapeutic benefits through reductions in mechanical allodynia, inflammation, and cartilage degradation in the context of osteoarthritis. While these preliminary findings suggest potential symptomatic and structural benefits, further studies are required to confirm therapeutic efficacy and clarify underlying mechanisms. To our knowledge, there is no other study examining the anti-arthritic effects of whole blueberries using this animal model.

Pain is one of the main symptoms of osteoarthritis. Compared to other symptoms, such as stiffness and disability, pain is more common in osteoarthritis [30]. It is multifactorial and is linked to both the peripheral and central nervous systems [30,31,32]. Pain is normally localized in the affected joint of patients with osteoarthritis. However, research has shown that pain sensitivity [33] and nociception [27] are not limited to the affected joint and are increased in other areas of the body [27,33]. Research has reported a link between cytokines and osteoarthritis pain [34,35]. Schaible (2012) states that some cytokines are pronociceptive and that they affect nociceptive neurons [35]. Cytokines TNFα and IL-6, when injected into normal knee joints, induced mechanical sensitization. However, neutralizing both TNFα and IL-6 inhibited the development of mechanical sensitization [35]. A study examining the association between IL-6 and central sensitization and pain behavior as a result of osteoarthritis reported the increased expression of spinal IL-6 in an MIA animal model. However, inhibiting the IL-6 receptor decreased pain hypersensitivity in the animals [36]. Also, in a clinical study, the level of IL-6 in synovial fluid was correlated with pain scores reported by patients with acute knee pain [34]. Therefore, examining the effects of specific cytokines on pain sensitization in osteoarthritis conditions could be essential in targeting and treating pain symptoms in osteoarthritis.

In addition, osteoarthritis is characterized by low-grade inflammation, which contributes to cartilage degradation and joint destruction [37]. The role of inflammation as an initiatory factor, as opposed to being a consequence of osteoarthritis disease, is still uncertain [37]. However, research studies have shown the linkage between inflammation and the progression of this condition [29,37,38]. During disease progression, macrophages produce cytokines that increase the production of metalloproteinases and other degradative mediators [37]. Both TNFα and IL-1β are considered key inflammatory mediators in the pathogenesis of osteoarthritis. The actions of these cytokines are similar and result in the activation of the same signaling pathways, which increase inflammation and joint damage [11]. TNFα is one of the many ligands in the TNF superfamily. Also, tumor necrosis factor receptor superfamily member 1 1β (Tnfrsf1 1β) is linked to chondrocyte formation, and its expression is elevated in patients with osteoarthritis [39]. Independently and in conjunction with other inflammatory mediators, IL-1β can induce inflammatory effects that cause joint destruction [11]. Some inflammatory cytokines act by stimulating cells to synthesize inflammatory mediators, thereby exhibiting autocrine or paracrine actions in inflammation. The proinflammatory cytokine IL-1β is one such mediator able to promote its own production. Osteoarthritis patients show increased concentrations of IL-1β in synovial fluid, cartilage, and subchondral bone [11]. IL-1β is activated when it binds to its receptor (IL-1R1). The activation of IL-1β initiates a cascade of signaling pathways and downstream targets that results in increased inflammation [11]. One such downstream target is TAK1, which interacts with IκK kinase (IKK) phosphorylation and, as a result, activates NFκB. The NFκB signaling pathway is responsible for the regulation of mediators that are directly involved in inflammation [15,40].

A previous study reported the decreased activation of NFκB signaling pathways in blueberry-fed mice [41]. Similarly, Vendrame et al. (2013) reported the decreased expression of NFκB with 8% whole blueberry in the diet of obese rats [19]. The decreased expression of NFκB may be attributed to its decreased activation. In a study examining the effect of blueberry extract on colitis, the results showed decreased activations of NFκB [42]. In colonic tissue, the notable phosphorylation of p65 NFκB was observed. However, the blueberry extract significantly decreased p65 NFκB activation in the colonic samples that were pretreated or treated with the blueberry extract [42]. Therefore, the anti-arthritic and anti-inflammatory effects of whole blueberries may be attributed to the inhibition of NFκB signaling pathways by suppressing IL-1β production, thereby preventing the release of other proinflammatory mediators.

Bone remodeling is another symptom linked to osteoarthritis. The subchondral bone in patients is weak as a result of decreased mineralization and increased collagen metabolism [43]. However, Couchourel et al. (2009) report that, in osteoarthritis, type 1 collagen exhibits higher expression, which should increase mineralization [44]. For type 1 collagen to successfully mineralize, its arrangement and ratio must be exact [43,44]. One study found increased mRNA expression in collagen type I chains, Col1a1 and Col1a2, in the hip samples of osteoarthritis patients compared to control non-osteoarthritis samples [43]. They reported a twofold increase in the mRNA ratio of Col1a1/Col1a2 in osteoarthritis bone in comparison to the control [43]. Another study found a 3.4-fold increase in the mRNA expression of Col1a1 in osteoarthritis osteoblasts in comparison to normal osteoblasts [44]. However, there was no change in the mRNA expression of Col1a2 [44]. Correspondingly, a previous study reported that inflammation, pannus formation, cartilage damage, and bone resorption were significantly inhibited in rats treated with 120 mg/kg of polyphenolic-enriched red raspberry extracts in an antigen-induced rat model [24]. Likewise, Figueira et al. (2016), using a collagen-induced arthritis rat model, reported decreased edema formation and improved histological damage and radiographic scores in the knee joint and hind paw of rats treated with a blueberry extract [16]. In addition, Li et al. (2014) investigated the therapeutic benefits of rabbiteye blueberries on osteoporosis using an ovariectomized rat model [22]. Similarly to the present study, they used a 10% dose; however, the freeze-dried powder was administered in a water solution by gavage. Their work demonstrated that dietary supplementation with rabbiteye blueberries significantly prevented bone loss in ovariectomized rats, as evidenced by improved bone mineral density and microarchitecture [22]. In the present study, we observed a similar protective effect on bones, notably through the upregulation of Col1a1, a key marker of bone matrix formation. The findings from Li et al. (2014) support the hypothesis that bioactive compounds in blueberries enhance osteoblast activity and collagen production, which is consistent with the elevated Col1a1 expression observed in both models [22]. However, a previous study in an ovariectomized bone loss model suggests that blueberries protect against bone loss by reducing bone resorption [21]. The results from that study showed increased levels of ALP, COL, and TRAP in the ovariectomized group in comparison to the sham and ovariectomized groups receiving blueberry treatment, resulting in the suppression of bone turnover [21]. Overall, these studies showed the potential protective effects of blueberries on bone loss and osteoporotic changes associated with estrogen deficiency in an ovariectomized rat model of osteoporosis [21,22]. Furthermore, there is a need for further studies to elucidate the mechanisms by which blueberries impact bone loss and the effective dose of blueberries.

There is increased research interest in natural alternatives with less severe side effects to treat symptoms associated with osteoarthritis. Studies have examined the effects of various natural products, such as fruits, and their effects on pain symptoms in in vivo and clinical studies [45]. Research shows the potential antinociceptive effects of fruits and bioactive compounds, such as strawberries [46], apples [47], resveratrol [28], and grape seed extracts [48]. Resveratrol, a natural antioxidant, was used to treat MIA-induced animals. The study examined the effects of resveratrol on mechanical, heat, and cold hyperplasia. The study demonstrated significantly decreased paw withdrawal time for mechanical, heat, and cold hyperplasia in the MIA + vehicle group compared to the control group on days 7 and 14 of treatment. Conversely, treatment with resveratrol increased the paw withdrawal time [29]. In addition, research examining the effect of *Arrabidaea chica* extracts showed a significant decrease in allodynia 21 days post-treatment [49]. Likewise, Yan et al. (2018) reported a significant reduction in mechanical allodynia thresholds after osteoarthritis induction compared to a control [50].

Natural products such as fruits and vegetables have shown promising health benefits when consumed and may be potentially beneficial in inhibiting symptoms associated with osteoarthritis [51]. Basu et al. (2018) state that berries contain a rich source of phytochemicals, such as polyphenols [2]. The phytochemicals enhance the positive health effects of fruits, thus making them potential therapeutic agents for diseases such as osteoarthritis [2]. Berries such as raspberries, strawberries, and blueberries have been reported to elicit positive effects against arthritis [2]. Additionally, blueberries have high phenolic content, which has been linked to their biological activity and numerous health benefits [16]. Therefore, the consumption of blueberries may provide a natural, complementary therapeutic agent in treating symptoms associated with osteoarthritis.

One potential limitation of the present study is attributed to the small sample size. Ten animals per group were divided between different outcome analyses, resulting in a smaller sample size for various end-point analyses. Although some outcomes failed to achieve significance, they were trending in a positive direction, indicating a potential effect of the whole-blueberry treatment. Also, another possible limitation of this study is the clinical translatability of the in vivo study design. The model used younger and lean rats for this study; however, research shows that two of the major risk factors for OA are age and obesity. But using younger samples allows us to control age- and obesity-related confounding variables, thereby providing a more uniform baseline for evaluating the direct impact of the intervention. Unfortunately, there is no animal model that will reflect all aspects of osteoarthritis in humans. The selected model mimics the key features of osteoarthritis pathophysiology and inflammatory pain mechanisms, which allows researchers to investigate potential therapeutic strategies that warrant further research. Thus, the preclinical osteoarthritis model provides an excellent opportunity to investigate specific pain mechanisms and osteoarthritis-related symptoms and examine therapeutic targets that are otherwise not possible in clinical approaches. Further studies are warranted to examine the effect of whole-blueberry treatment on osteoarthritis in clinical trials.

## 5. Conclusions

The MIA-induced model mimics features that are representative of the degradation observed in clinical osteoarthritis. The results of this study demonstrated increased inflammation and cartilage degradation consistent with the proposed model. Within this disease condition, treatment with whole blueberries and their bioactive compounds demonstrated protective effects. The positive effects involved antinociceptive and anti-inflammatory actions and improved structural integrity within the joint. In the present study, we examined the effects of whole blueberries in MIA-induced rats. While some parameters displayed improvements, these effects were not consistently statistically significant. In addition, a clear dose-dependent response was not observed. The preliminary findings indicate the potential biological effects of blueberries on osteoarthritic outcomes, but further studies are warranted to confirm efficacy and elucidate mechanisms. Overall, the results of this study provide insights regarding the therapeutic potential of whole blueberries as a modality for treating osteoarthritis.

## Figures and Tables

**Table 1:** Dietary treatments of whole blueberries in MIA-induced and control rats.

Groups	Treatment Group *(n* = 10)	MIA Injection	Protein Source	Blueberry (BB) (g/kg Diet)
1	Non-MIA (Control)	No	Casein-Based	0
2	MIA	Yes	Casein-Based	0
3	MIA-5% BB	Yes	Casein-Based	50
4	MIA-10% BB	Yes	Casein-Based	100

All animals received the dietary interventions for 48 days. Each treatment group consisted of 10 animals.

**Table 2:** Histopathological analysis criteria.

Parameters	Descriptions	Score
Inflammatory Infiltration	Normal, no infiltrate present.	0
	Minimal infiltrate detected.	1
	Mild infiltrate present.	2
	Moderate infiltrate present with loss of synovial structure.	3
	Severe infiltrate present with loss of synovial and articular structure.	4
Synovial Hyperplasia	Normal, none present.	0
	Minimal synovial hyperplasia detected.	1
	Mild synovial hyperplasia.	2
	Moderate synovial hyperplasia.	3
	Severe synovial hyperplasia present with loss of synovial structure. Expansion of synovial lining.	4
Erosion of the Cartilage and Bone	Normal, no erosion detected.	0
	Minimal erosion detected in fibrillation of the cartilage.	1
	Mild erosion cartilage loss and mild erosion of the subchondral bone.	2
	Moderate cartilage fibrillation and loss with infiltration of the subchondral bone.	3
	Severe cartilage and bone erosion present with loss of cartilage and infiltration of the bone.	4

Stained joint sections were examined and scored for inflammatory infiltration, synovial hyperplasia, and the erosion of the cartilage and bone. Each joint section was scored on a scale of 0 to 4 according to previous studies [24,25].

**Table 3:** Effects of whole-blueberry treatment on joint diameter (as a measure of edema) across the treatment and control groups.

Diameters (mm) of the Joint								
Groups	Day 0		Day 13		Day 26		Day 40	
	Ipsi	Contra	Ipsi	Contra	Ipsi	Contra	Ipsi	Contra
Control	6.0 ± 0.3	4.8 ± 0.3	5.7 ± 0.3	5.3 ± 0.3	4.5 ± 0.2	4.5 ± 0.2	5.7 ± 0.2	5.3 ± 0.2
MIA	6.0 ± 0.4	4.3 ± 0.2	5.7 ± 0.3	5.2 ± 0.5	5.5 ± 0.2	5.2 ± 0.4	6.0 ± 0.3	5.0 ± 0.3
MIA + 5% BB	5.8 ± 0.3	5.0 ± 0.4	5.8 ± 0.3	5.2 ± 0.3	5.0 ± 0.4	4.5 ± 0.2	5.8 ± 0.2	5.0 ± 0.3
MIA + 10% BB	6.0 ± 0.4	5.2 ± 0.6	6.2 ± 0.4	5.0 ± 0.4	6.0 ± 0.4	5.2 ± 0.4	5.7 ± 0.2	5.3 ± 0.2

Measurements were taken at baseline, day 13, day 26, and day 40 of the treatment. N = 6 for each group. The results shown are expressed as mean ± SEM, *p* ≤ 0.05. Ipsi: Ipsilateral (right joint); Contra: contralateral (left joint).

## Data Availability

The original contributions presented in this study are included in the article. Further inquiries can be directed to the corresponding author.
